# Deletion polymorphism at chromosome 3q26.1 and oral squamous cell carcinoma

**DOI:** 10.3892/ijo.2012.1749

**Published:** 2012-12-21

**Authors:** HOMARE KAWACHI, KEISUKE SUGAHARA, YASUTAKA NAKAMURA, AKIRA KATAKURA, KIYOSHI MINAGUCHI, TAKAHIKO SHIBAHARA

**Affiliations:** 1 Departments of Oral and Maxillofacial Surgery, Tokyo Dental College, Tokyo, Japan; 2 Forensic Odontology, Tokyo Dental College, Tokyo, Japan; 3 Oral Medicine, Tokyo Dental College, Tokyo, Japan

**Keywords:** oral squamous cell carcinoma, 3q26.1, deletion, polymorphism, real-time PCR

## Abstract

Several recent studies have investigated DNA instability in malignancies including deletions and duplications of part of the chromosome using array-based comparative genomic hybridization (CGH) analysis. Using the same approach on oral squamous cell carcinoma (OSCC) tissue samples, we found a frequent deletion at chromosome 3q26.1 in OSCC patients; this polymorphism showed a gene frequency of 0.293–0.368 in healthy volunteers (n=60) and 0.129–0.195 in OSCC patients (n=54). Detailed analysis around the polymorphic region revealed the deletion breakage point. A significant association of gene frequency for the deletion polymorphism between healthy volunteers and patients implicated genetic factors related to this polymorphism in the development of OSCC. Currently, no gene is predicted to lie within the 3,606-kbp region around the polymorphism. Thus, although a single-gene model could not explain the occurrence of OSCC, we believe that examining this polymorphism could be useful in identifying risk factors for OSCC.

## Introduction

Recent years have seen an increased incidence of oral squamous cell carcinoma (OSCC) cases in Japan, coinciding with population aging. OSCC accounts for 1–2% of all cancers, and approximately 40% of head and neck cancers. The ratio between men and women for OSCC is 3:2. Globally, the prevalence of OSCC is high in nations with high levels of alcohol and tobacco use (1–3). Pathologically, 80% of oral cancers are squamous cell carcinoma (4,5).

Like many other cancers, the occurrence of OSCC is thought to be intricately associated with both genetic and environmental factors. As the entry of the gastrointestinal system, the oral cavity is exposed to various environmental insults such as chemical stimuli from alcohol and tobacco (6,7), food, and physical stimuli from dental caries and faulty dental prostheses. All such insults are considered carcinogenic risk factors for the oral mucosal membranes (8–11).

A review of chromosomal aberrations in oral or head and neck squamous cell carcinoma concluded that the most significant findings are chromosomal changes, suggesting the involvement of tumor suppressor genes (TSGs) (12). In studying the relationship between OSCC metastasis and chromosomal aberration, we found frequent deletion of a probe on chromosome 3 by array-based comparative genomic hybridization (CGH) analysis of OSCC tissues. The deletion was not related to metastasis (13). The present study further examined how the identified chromosomal deletion relates to OSCC.

## Materials and methods

### Samples

Twenty tumor tissue samples were used for extraction of DNA for array-based CGH analysis and also for real-time PCR. DNA for real-time PCR was also collected from another 11 tumor tissues, 31 marginal tissues around the tumors, and 31 peripheral blood samples from healthy volunteers. A total of 54 and 60 peripheral blood samples were further obtained from different OSCC patients and healthy volunteers, respectively. All OSCC patients underwent surgical resection at Tokyo Dental College, Chiba, Japan, between April 2007 and July 2010. Healthy volunteers were selected from workers at the same college. Written informed consent was obtained from all participants in accordance with the Ethical Guidelines on the Use of Human Tissues. The Ethics Committee of Tokyo Dental College approved the study (approval no. 205). The 54 OSCC patients were aged from 43 to 89 years, and the 60 healthy volunteers were aged from 24 to 48 years. Table I summarizes the clinical characteristics of the study participants.

### DNA extraction

Sample for DNA extraction were stored at −20°C until use. Genomic DNA was extracted from peripheral blood using a QIAamp DNA blood midi kit (Qiagen, Valencia, CA) and from tissues using a QIAamp DNA Maxi kit (Qiagen) according to the instructions supplied by the manufacturer. The DNA was quantified using a Nano drop^®^ (ND-1000 Spectrophotometer, Thermo Fisher Scientific, Waltham, MA).

### Array-based CGH analysis

Array-based CGH analysis was performed on primary tumor tissues using a Human Genome CGH Microarray Kit 44K (Agilent Technologies, Santa Clara, CA), containing *in situ*-synthesized 60-mer oligonucleotides representing 42,494 unique probes for human genes. Labeling and hybridization were performed essentially as described previously with the following modifications. We amplified 100 ng each of reference (male or female human genomic DNA; Promega, Madison, WI) and tumor DNA with Phi29 DNA polymerase according to the protocols provided by the supplier (Qiagen). DNA was then digested with *Alu*I (50 units) and *Rsa*I (50 units; Promega) for 2 h at 37°C. Digests were filtered using the QIAprep Spin Miniprep kit (Qiagen) and then verified on a Bioanalyzer (Agilent Technologies). Fluorescent labeling reactions to make hybridization probes were performed with 7 *μ*g of purified restricted DNA using a BioPrime array-based CGH genomic labeling kit (Invitrogen), according to the instructions provided by the manufacturer, in a volume of 50 *μ*l with a modified dUTP pool containing 120 *μ*M each of dATP, dGTP, and dCTP; 60 *μ*M dTTP; and 60 *μ*M Cy5-dUTP or Cy3-dUTP (Perkin-Elmer, Waltham, MA). Labeled reference and tumor DNA probes were subsequently mixed and filtered through a Microcon YM-30 column (Millipore, Billerica, MA) then verified on a Bioanalyzer (Agilent Technologies). To the mixtures were added 50 *μ*g of Human Cot-1 DNA (Invitrogen)/Agilent 10X Blocking Agent/Agilent 2 Hybridization Buffer. Before hybridization to the array, the hybridization mixtures were denatured at 95°C for 3 min, and then incubated at 37°C for 30 min. The mixtures were centrifuged at 17,900 × g for 1 min to remove any precipitate, and then applied to the array using an Agilent microarray hybridization chamber. Hybridization was carried out for 40 h at 65°C in a rotating oven (Robbins Scientific, Mountain View, CA) at 20 rpm. The arrays were then disassembled in 0.5X SSC/0.005% Triton X-102 at room temperature, washed for 5 min at room temperature in wash 1, and then incubated for 1 min at 37°C in 0.1X SSC/0.005% Triton X-102 (wash 2). Slides were dried and scanned using an Agilent G2565B DNA microarray scanner (14,15).

### Real-time PCR

Real-time PCR was performed using tumor, marginal tissue, and blood samples with SYBR Green I fluorescence detection on a Light Cycler 480 (Roche Diagnostics, Basel, Switzerland). Oligonucleotide primers for real-time PCR were designed using Primer software (Whitehead Institute for Biomedical Research), and uniqueness in the human genome was checked by a BLAST search. Nucleotide sequences of oligonucleotide primers were: F, 5′-CGGGGAGTTTGATTTTCACT-3′ and R, 5′-CGCTGTATGGTTGTCTTGTTG-3′. The 20-*μ*l reaction mixture consisted of 10 *μ*l 2X IQ SYBR Green Supermix (Bio-Rad Laboratories, Hercules, CA), 2.5 ng genomic DNA, and 800 nM of each PCR primer. The reaction mixtures were heated at 95°C for 10 min and then subjected to 40 rounds of two-step temperature cycling (95°C for 15 sec and 65°C for 60 sec) (16). The crossing point for each amplification curve was determined by the second derivative maximum method. The standard curve method using separate reaction wells was applied for relative quantification. We used NAGK (N-acetyl glucosamine kinase, NAGK) as internal reference loci (17).

### Detection method for comparison and typing of polymorphic regions

Table II details the PCR primer sequences used for amplifying the regions around the hybridized probe sequence by array-based CGH and the PCR target sequences. PCR amplification was performed in a 30-*μ*l mixture containing 10 ng genomic DNA, 10 mM Tris-HCl at pH 8.3, 50 mM KCl, 2.5 mM MgCl_2_, 0.02% gelatin, 200 *μ*M dNTP, 400 nM of each primer, and 1.25 U AmpliTaq Gold (Applied Biosystems). A two-step PCR amplification process was used: 95°C for 11 min, followed by 28 cycles of denaturation at 95°C for 40 sec, and annealing and extension at 59°C for 105 sec. After the 28th cycles, a final extension step was performed at an annealing/extension temperature at 60°C for 10 min. PCR products were separated on 8% polyacrylamide gels, and all products were visualized by silver staining (18).

### Sequence analysis

PCR for sequencing was performed using the BigDye™ Terminator v1.1 Cycle Sequencing Ready Reaction Kit (Applied Biosystems), and the products were purified using a PCR purification kit (Invitrogen). Excess dye was removed using Performa DTR gel filtration cartridges (EdgeBio, www.edgebio.com). Sequence analysis was performed on an ABI 3100 DNA sequencer.

### Statistical analysis

The χ^2^ test was used for comparisons between the presence and absence of deleted regions, using a 2×2 contingency table. The homogeneity test for gene frequencies was performed according to Samaneh *et al*(2010)(19).

## Results

### Array-based CGH analysis

**Figure 1.**
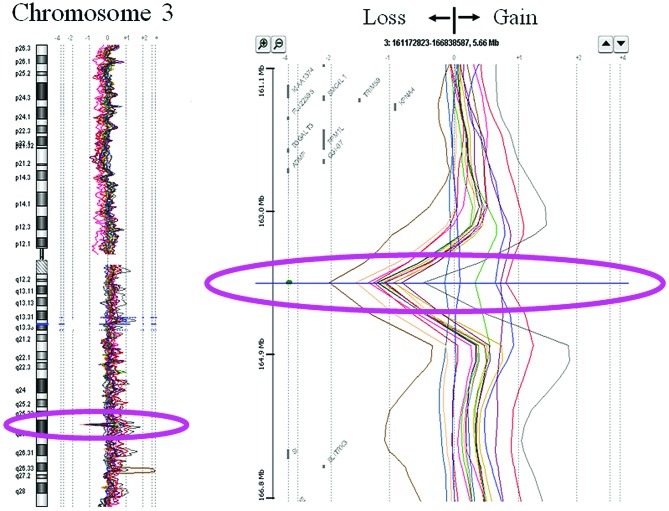
Schematic illustration of cytogenetic alteration in chromosome 3q in OSCC. DNA obtained from OSCC tissues samples. The portion of chromosome 3q26.1 was deficient in 14 of 20 people (70%).

The array-based CGH analysis was performed on 20 OSCC tumor tissue samples using Feature Extraction software (version 8.5.1.1, Agilent Technologies) and the linear normalization method for background subtraction. Datasets created from the dye-swap experiments were averaged and then further analyzed using CGH Analytics software (version 3.4, Agilent Technologies). Although we were originally investigating the relationship between metastasis and chromosomal aberration, a frequent deletion on chromosome 3 showing up on the array-based CGH analysis was not associated with any clinical features of metastasis (13). This deletion was found in 70% (14/20) of DNA extracts from OSCC tissues samples on the long arm of chromosome 3 (Fig. 1). Detailed analysis mapped the region to chromosome 3q26.1. Because the deletion frequency was so high in OSCC tissues, we next studied its relationship to other clinical features.

### Examination of the deleted region by real-time PCR

We next applied real-time PCR to further study the deleted region at chromosome 3q26.1 identified by array-based CGH (array-based CGH probe sequence = 3q26.1-pro-seq). Real-time PCR primers were initially constructed to amplify the probe region, 5′-flanking region, and 3′-flanking region of the probe. However, because the most similar results to those on array-based CGH analysis were obtained using primers for the 5′-flanking region, we used these primers for further studies. DNA samples were isolated from another 11 OSCC tissues and from marginal tissues around the tumor in the 31 OSCC patients. When real-time PCR was conducted for DNA isolated from tumor and marginal tissues, the same tendency for quantification was obtained from both sample types. Assuming that marginal tissues contained mainly normal tissue, the real-time PCR suggested that the deletion of 3q26.1-pro-seq is not due to chromosomal aberration resulting from carcinogenesis, but instead occurs through inherent genetic variation.

**Figure 2.**
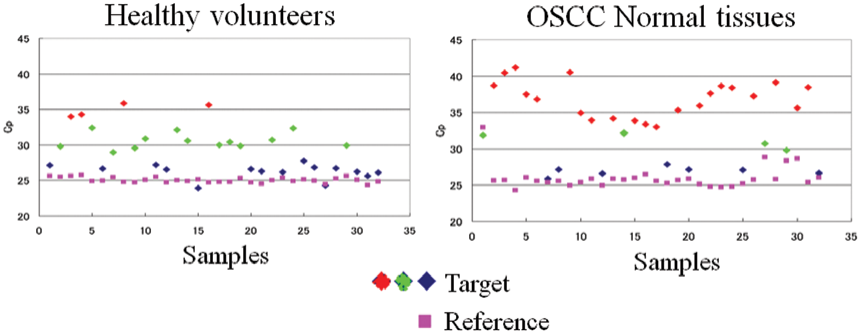
Schematic representation of the DNA copy number differences between blood sample from a healthy volunteer and normal tissues of a patient with OSCC, detected at 3q26.1 by real-time PCR. Pink, reference primer. Red, green, and blue bands, target primers.

To explore if the deletion around 3q26.1-pro-seq is genetically restricted to OSCC patients, we collected DNA samples from 31 healthy volunteers as controls and performed real-time PCR for the 5′-flanking region of 3q26.1-pro-seq (Fig. 2). If the deletion was indeed genetic variation, the real-time PCR results must be classified into three types: complete deletion, hemizygote, and non-deletion homozygote. Although the distribution of Cp values could not be clearly divided, we classified them into three groups using tentative threshold values. The average Cp values for these groups were 26.5±2, 30.5±2, and 34.5±2, respectively. The first type corresponded to homozygotes for 3q26.1-pro-seq (+), the second type to heterozygotes for 3q26.1-pro-seq, and the third type to 3q26.1-pro-seq (−) homozygotes. Because the boundary lines of these values were not clear-cut, we classified them into 3q26.1-pro-seq-positive type (+) (non-deletion homozygote and heterozygote) and 3q26.1-pro-seq-loss type (−), to compare frequencies between healthy and OSCC patient groups by the χ^2^ test (Table II). A statistically significant difference was observed regarding presence and loss of 3q26.1-pro-seq between the OSCC patients and healthy volunteers (P=8.1×E^−06^; Table II). Loss of 3q26.1-pro-seq was more common in OSCC patients.

### Detection and evaluation of genetic variation

Although significant association was observed in the distribution of 3q26.1-pro-seq-positive and -negative individuals, the total number of samples was small (31 per group). In addition, the real-time PCR typing results were ambiguous because the boundaries between genotypes were unclear. We therefore sought to establish reliable typing methods and increase the number of samples to confirm if the suspected association was conserved in new population samples.

**Figure 3.**
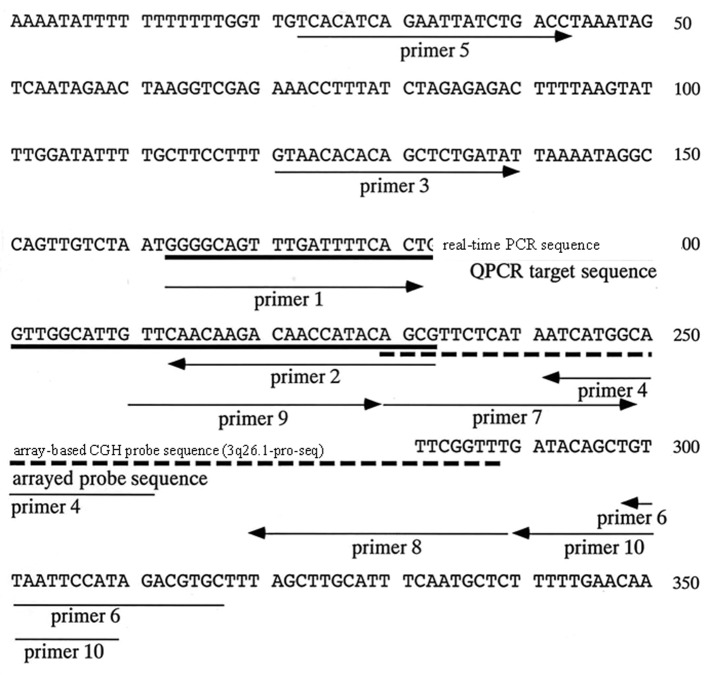
Nucleotide sequences around the real-time PCR target region and array-based CGH probe sequence. Primers (1–10) used to amplify various regions around the target regions are displayed, with arrows showing the 5′→3′ primer sequences.

We first compared the presence and absence of the 3q26.1-pro-seq sequence using simple PCR amplification followed by native polyacrylamide gel electrophoresis (Fig. 3). First, we constructed primers (Fig. 3, primer 3–4) to amplify the fragment (141-bp fragment) that included the target region for real-time PCR (71-bp fragment amplifiable by primer 1 and 2) (Fig. 3). When a 141-bp fragment was amplified by PCR in 35 cycles of amplification including 2.5 U of AmpliTaq Gold, strong positive bands or faint bands corresponding to the same migration position as the target PCR product appeared in many samples, and negative samples were rare. Sequencing of the faint band and the intensified band determined that the amplified fragments were identical. National Center for Biotechnology Information (NCBI) blast searching using primers 3 and 4 revealed no similar sequences to the 141-bp fragment on chromosome 3. This suggested that the faint band was not amplified from different similar sequences in the genome, and contamination with foreign DNA occurred in the course of DNA collection and experiments. All reagents used for the PCR amplification were subsequently replaced to identify the possible source of contamination. Indeed, we employed a different company to synthesize the oligonucleotide primers used to amplify the 141-bp products. However, similar results were obtained, suggesting that the contamination did not occur during PCR amplification.

Next, we constructed primers to amplify a large fragment (294-bp) including the 141-bp fragment and 3q26.1-pro-seq to ascertain whether the unstable amplification among samples was due to sequence heterogeneity around the 141-bp fragment (Fig. 3). PCR amplification was then performed using three different sets of primers that each amplified the 141-bp fragment, the real-time PCR fragment (designated as a 71-bp fragment amplified by primer 1–2 in the following explanation), and the 294-bp fragment (Fig. 3 and Table III). The amplified products were not always present depending on the samples examined. Therefore, we could not determine a reliable sample genotype at this stage.

To solve this problem, we constructed two further primers; one to amplify the 3q26.1-pro-seq (60-bp fragment amplified by primer 7–8; Fig. 3 and Table III) and the other to cover the 3q26.1-pro-seq region (108-bp amplified by primer 9–10; Fig. 3 and Table III). These two primers were also used for typing together with the three previous primer pairs. In short, five kinds of PCR amplification, all producing the 141-, 71-, 294-, 60-, and 108-bp fragment, were performed for each sample to compare presence and absence of the PCR product. Again, the samples were heterogeneous with respect to the five fragments always being present, and amplification efficiency of the five fragments continued to vary among samples. We therefore arranged the method of PCR amplification as shown in Materials and methods; such that the number of PCR amplification cycles, volumes of Taq polymerase, and primer concentrations were decreased. This change in PCR conditions decreased the number of faint bands after amplification, and increased the reproducibility of amplification efficiency from the same samples. Intensity of the positive amplification products became similar among positive samples, and delicate faint bands decreased considerably in negative-like samples. This PCR amplification protocol was subsequently used to type new random samples.

**Figure 4.**
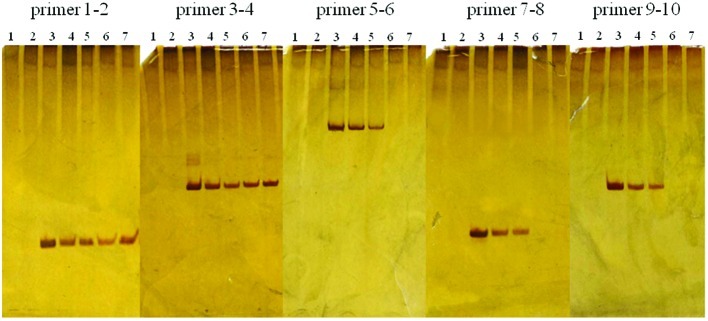
DNA polymorphism analysis. Native-gel electrophoresis after silver staining. Channels 1–7 show electrophoresis from different individuals. DNA samples from seven individuals were amplified using primer pairs 1–2, 3–4, 5–6, 7–8, and 9–10. Channel 1 and 2 are negative for all primer pairs. Channels 3, 4, and 5 are positive for all primer pairs. Individuals 6 and 7 are positive for primer pairs 1–2 and 3–4, but negative for primer pairs 5–6, 7–8, and 9–10.

We then typed 60 healthy volunteers and 54 OSCC patients. Final typing for positive (+) or negative (−) was determined by the presence or absence, respectively, of a distinct band. When a faint band was observed, PCR amplification was performed two more times to determine if the same result could be obtained. When a faint band was reproduced three times, we considered the sample as positive (+), but marked that the band intensity was weak. When the faint band was not reproducible, it was described as such. The results of typing are shown in Fig. 4 and Table IV. Identical results were obtained with the five different PCR products in most subjects (89% of 114 subjects). However, 6 samples from each group (healthy individuals and OSCC patients) showed variation among the different PCR products. They were (+) for a 71-bp product (produced by primer 1–2) and the 141-bp product (produced by primer 3–4), but (−) for the 294-, 60-, and 108-bp products (produced by 5–6, 7–8, and 9–10, respectively) (Table II). Among these samples, 2 out of 6 (−) samples in the OSCC patient group showed amplification of 71- and 108-bp products as faint bands in the three PCR trials. Finally, the 12 variable samples were further amplified using three primer pairs, 1–4, 1–8, or 1–10. All but one of the samples was (+) for the products amplified by primer pair 1–4, but negative for those by primer pairs 1–8 and 1–10. The exception was obtained from an OSCC patient, who was (−) for all primer pairs (Table IV).

### Comparison of gene frequencies between healthy volunteers and OSCC patients

As mentioned above, typing of each individual was different depending on the primers used. Because this variability could be due to genetic polymorphism, gene frequency was estimated in the healthy individuals and OSCC patients. Different genes were hypothesized depending on the size of fragments. The gene frequencies were estimated by the presence and absence of the 71-bp fragment produced by primer pair 1–2 and the 108-bp fragment produced by primer pair 9–10. Because we could not discriminate homozygote and heterozygote by the present method, we regarded (+) individuals as a dominant type composed of homozygote (+)/(+) and heterozygote (+)/(−) types, and (−) individuals as a recessive genotype composed of negative homozygotes (−)/(−).

Gene frequencies for the 71-bp fragment were 0.368±0.050 and 0.632±0.050 for the 71-bp (+) and (−) genes, respectively, in the healthy population, with 0.195±0.052 and 0.805±0.052 calculated as the frequencies of the 71-bp (+) and (−) genes, respectively, in the OSCC patient population. This difference in gene frequencies between healthy and OSCC patient populations was significant (χ^2^=8.449, df=1, 0.01<P<0.001).

Gene frequencies for the 108-bp fragment were 0.293±0.046 and 0.707±0.046 for the 108-bp (+) and (−) genes, respectively, in the healthy population, with 0.129±0.033 and 0.871±0.033 for the 108-bp (+) and (−) genes, respectively, in the OSCC patient population; also a significant difference (χ^2^=9.209, df=1, 0.01<P<0.001). These results suggested that genetic variation of a region at chromosome 3q26.1 is associated with OSCC, and absence of this region was more common in OSCC patients.

## Discussion

In the present study, we used a simple method for PCR amplification and detection by native gel electrophoresis to detect genetic variation of the polymorphic region found previously on chromosome 3q26.1 (3q26.1-pro-seq) in OSCC samples. To confirm the presence or absence of the region by PCR, we designed primers to amplify larger fragments containing the region identified by real-time PCR. We expected apparent variation in the presence or absence of amplified products irrespective of the difference in the positive homozygote or hemizygote pattern. However, amplification was not stable even between replicates of the same samples. We first suspected contamination occurring during the PCR amplification or genomic DNA isolation steps, and took steps to eliminate this possibility. We changed all reagents used for PCR amplification, conducted dual PCR of the blind PCR products, and synthesized new primers at a different company. However, contamination was suspected only in the process of dual PCR amplification of control water solution in some experiments. In these cases, contamination was not detected in the first PCR amplification. We then isolated DNA from selected subjects by the classical method of ethanol precipitation to test for contamination with foreign DNA, but comparison of PCR amplification profiles for the 141-bp fragment showed no difference between samples isolated by the two procedures. We concluded that contamination was not responsible for the variable amplification of faint bands in the present study.

We therefore decided to control the method of PCR amplification to avoid excessive amplification of the PCR products. Although approximately 90% of the samples showed clear-cut results concerning presence or absence of the five different-sized PCR products among samples from the same subjects, approximately 10% of the subjects showed different PCR amplification profiles depending on the primer pairs used for amplification. Faint and unstable PCR products are amplified when highly degraded or small amounts of DNA are used as templates for PCR amplification in common forensic cases or when primer sequences do not match sufficiently. We therefore concluded that in some of the deletion genotypes, breakage points might exist near the 3q26.1-pro-seq, with the most possible region covered by primer 4–10. In addition, breakage points may not always be identical among individuals, because amplification efficiencies differ among primer pairs. Based on these observations, we finally selected two regions (71-bp fragment amplified by primer pair 1–2 and 108-bp fragment amplified by primer pair 9–10) to determine presence or absence of the defect region in this study.

Significant differences were observed in the frequencies of polymorphism of the region around the 3q26.1-pro-seq between the healthy volunteers and OSCC patients, suggesting that a deletion polymorphism in this region (chromosome 3q26.1) could be associated with occurrence of OSCC. We also tested the association between presence and absence of the amplified products and clinical characteristics of OSCC patients. However, no statistically significant association was observed.

The 3q26.1 region was previously reported to harbor colorectal cancer susceptibility loci in earlier studies, but no candidate gene was identified. This region contains an element that regulates the expression of an upstream candidate tumor suppressor, PPM1L, thus providing a novel mechanism for colorectal tumorigenesis in APC mutation-negative familial colorectal cancer (20). Searches for a possible gene around the present deleted region using genome browser did not hit any candidates, and thus because there are reported defects around chromosome 3q26.1 in cancer patients, we cannot validate this association with OSCC in the present study due to insufficient patient numbers. Further studies involving large-scale sample collection and analysis are necessary to determine the reliability of our suggested association. The range of the deleted region should also be defined further to identify potential genes known to affect the development of OSCC.

## Figures and Tables

**Table I.** Clinical characteristics of OSCC patients and healthy volunteers.

	OSCC patients (n=54)

Age (years; means ± SD)	66±11.67
Gender	
Male	27
Female	27
Tobacco consumption	
Negative	22
Positive	32
Alcohol consumption	
Negative	32
Positive	22
Site	
Tongue	28
Gingiva	16
Palate	1
Buccal mucosa	5
Oral floor	4
T classification	
T1	18
T2	23
T3	5
T4	8
Stage classification	
I	16
II	16
III	9
IV	13

	Healthy volunteers (n=60)

Age (years; means ± SD)	29±4.57
Gender	
Male	46
Female	14
Tobacco consumption	
Negative	41
Positive	19
Alcohol consumption	
Negative	1
Positive	59

**Table II.** Gene polymorphism at chromosome 3q26.1 between healthy volunteers and OSCC patients by real-time PCR.

	Healthy volunteers	OSCC patients	P-value
Normal	27	10	<0.05
Loss	4	21	

**Table III.** Oligonucleotide sequences used for PCR.

Primer name	Type	Oligonucleotide sequence
1	Forward	5′-CGGGGAGTTTGATTTTCACT-3′
2	Reverse	5′-CGCTGTATGGTTGTCTTGTTG-3′
3	Forward	5′-GTAACACACAGCTCTGATAT-3′
4	Reverse	5′-GCCACTATAAATGCCATGAT-3′
5	Forward	5′-TCACATCAGAATTATCTGACC-3′
6	Reverse	5′-CACGTCTATGGAATTAACA-3′
7	Forward	5′-AGCGTTCTCATAATCATGGC-3′
8	Reverse	5′-AAACCGAAGTTAAGGAAAGT-3′
9	Forward	5′-GTTCAACAAGACAACCATAC-3′
10	Reverse	5′-ATGGAATTAACAGCTGTATC-3′

**Table IV.** Results of different sized PCR products.

Amplified product size (bp)	71	141	294	60	108	
Combination of primer no.	1–2	3–4	5–6	7–8	9–10	No. of samples
Healthy subject	(+)	(+)	(+)	(+)	(+)	30
	(+)	(+)	(−)	(−)	(−)	6
	(−)	(−)	(−)	(−)	(−)	24
OSCC subject	(+)	(+)	(+)	(+)	(+)	13
	(+)	(+)	(−)	(−)	(−)	6^a^
	(−)	(−)	(−)	(−)	(−)	35

a One of these samples were (−) for amplification by primer pair 1–8.
